# Formations of calcium carbonate minerals by bacteria and its multiple applications

**DOI:** 10.1186/s40064-016-1869-2

**Published:** 2016-03-01

**Authors:** Periasamy Anbu, Chang-Ho Kang, Yu-Jin Shin, Jae-Seong So

**Affiliations:** Department of Biological Engineering, Inha University, Incheon, 402-751 Republic of Korea

**Keywords:** Biomineralization, Calcite, CO_2_ sequestration, MICP, Urease, Urea hydrolysis

## Abstract

Biomineralization is a naturally occurring process in living organisms. In this review, we discuss microbially induced calcium carbonate precipitation (MICP) in detail. In the MICP process, urease plays a major role in urea hydrolysis by a wide variety of microorganisms capable of producing high levels of urease. We also elaborate on the different polymorphs and the role of calcium in the formation of calcite crystal structures using various calcium sources. Additionally, the environmental factors affecting the production of urease and carbonate precipitation are discussed. This MICP is a promising, eco-friendly alternative approach to conventional and current remediation technologies to solve environmental problems in multidisciplinary fields. Multiple applications of MICP such as removal of heavy metals and radionuclides, improve the quality of construction materials and sequestration of atmospheric CO_2_ are discussed. In addition, we discuss other applications such as removal of calcium ions, PCBs and use of filler in rubber and plastics and fluorescent particles in stationary ink and stationary markers. MICP technology has become an efficient aspect of multidisciplinary fields. This report not only highlights the major strengths of MICP, but also discusses the limitations to application of this technology on a commercial scale.

## Background

Biomineralization is the chemical alteration of an environment by microbial activity that results in the precipitation of minerals (Stocks-Fischer et al. 1999; Barkay and Schaefer 2001; Phillips et al. 2013). In nature, biomineralization is a widespread phenomenon leading to the formation of more than 60 different biological minerals (Sarikaya 1999) that exists as extracellularly inorganic crystals (Dhami et al. 2013a) or intracellularly (Konishi et al. 2006; Yoshida et al. 2010). Extracellular mineralization syntheses (for e.g., carbonate precipitation) from all groups of living organisms are widespread and well known phenomena (Lowenstam 1981). Most crystals formed through biomineralization consist of inorganic minerals, but they may also contain trace elements of organic compounds, which can regulate the biomineralization process (Yoshida et al. 2010). There are three different mechanisms involved in the production of biominerals: (1) Biologically controlled mineralization consists of cellular activities that specifically direct the formation of minerals (Lowenstam and Weiner 1989; Benzerara et al. 2011; Phillips et al. 2013). In this process, organisms control nucleation and growth of minerals. The minerals are directly synthesized at a specific location within or on the cell, but only under certain conditions. (2) Biologically influenced mineralization is the process by which passive mineral precipitation is caused by the presence of cell surface organic matter such as extracellular polymeric substances associated with biofilms (Benzerara et al. 2011; Phillips et al. 2013). (3) Biologically induced mineralization is the chemical modification of an environment by biological activity that results in supersaturation and the precipitation of minerals (Lowenstam and Weiner 1989; Stocks-Fischer et al. 1999; De Muynck et al. 2010; Phillips et al. 2013).

## Microbially induced calcite precipitation

Microbially induced calcite precipitation (MICP) refers to the formation of calcium carbonate from a supersaturated solution due to the presence of their microbial cells and biochemical activities (Bosak 2011). During MICP, organisms are able to secrete one or more metabolic products (CO_3_^2−^) that react with ions (Ca^2+^) in the environment resulting in the subsequent precipitation of minerals. Previously, the formation of calcium carbonate precipitation was proposed to occur via different mechanisms such as photosynthesis (Thompson and Ferris 1990; McConnaughey and Whelan 1997), urea hydrolysis (Stocks-Fischer et al. 1999; De Muynck et al. 2010; Dhami et al. 2013a), sulfate reduction (Castanier et al. 1999; Warthmann et al. 2000; Hammes et al. 2003a), anaerobic sulfide oxidation (Warthmann et al. 2000), biofilm and extracellular polymeric substances (Kawaguchi and Decho 2002; Arias and Fernandez 2008). However, the precipitation of calcium carbonate by bacteria via urea hydrolysis is the most widely used method (Hammes and Verstraete 2002; DeJong et al. 2010; De Muynck et al. 2010).

The ability of urease (urea amidohydrolase; EC 3.5.1.5) to induce carbonate precipitation in microorganisms has already been discussed by several researchers (Hammes et al. 2003a; Burbank et al. 2012; Li et al. 2013; Stabnikov et al. 2013). Urease activity is found in a wide range of microorganisms, but some strains produce particularly high levels of urease (Table 1). For example, *Sporosarcina pasteurii* (formerly *Bacillus pasteurii*) is a soil, non-pathogenic and endospore producing bacteria with an optimum pH for growth of 9.0 that can tolerate extreme conditions. Accordingly, many researchers have extensively studied use of this strain for MICP (Bang et al. 2001; Hammes et al. 2003a; Mitchell and Ferris 2006; Achal et al. 2009a; Okwadha and Li 2010; Tobler et al. 2011; Cuthbert et al. 2012; Qabany et al. 2012; Gorospe et al. 2013). Additionally, Achal et al. (2009a) developed a mutant strain (BP-M-3) of *Sporosarcina pasteurii* MTCC 1761 that produced an enhanced level of urease activity and calcite precipitation compared to the wild type. Some pathogenic bacteria such as *Helicobacter pylori, Proteus vulgaris, Staphylococcus aureus* and *Pseudomonas aeruginosa* also produce urease, during urinary infection and are involved in the formation of intracellular urinary stones (Hesse and Heimbach 1999; Stabnikov et al. 2013).

**Table 1:** Urease producing bacteria from various sources and amount of urease activity and calcite precipitation

Bacteria	Isolation site	Urease activity	Calcite precipitation	References
*Bacillus* sp. CR2	Mine tailing soil Urumqi, China	432 U/ml	2.32 mg/cell mass (mg)	Achal and Pan (2014)
*L. sphaericus* CH5	Abandoned express way and abandoned mining sites, Gangwondo, Korea	–	980 mg/100 ml	Kang et al. (2014a)
*Sporosarcinapasteurii*	Phenotypic mutant strain	550 U/ml	–	Achal et al. (2009a)
*B. pasteurii* NCIM 2477	Culture obtained from NCIM, Pune, India	18 U/ml	–	Sarada et al. (2009)
*K. flava* CR1	Mining ore soil, Urumqi, China	472 U/ml	–	Achal et al. (2011)
*B. megaterium*SS3	Calcareous soil, Andhra Pradesh, India	690 U/ml	187 mg/100 ml	Dhami et al. (2013b, 2014)
*B. thuringiensis*	Calcareous soil, Andhra Pradesh, India	620 U/ml	167 mg/100 ml	Dhami et al. (2013b)
*Halomonas*sp. SR4	Mine tailing, China	374.5 U/ml	–	Achal et al. (2012a, b, c)

**Fig. 1:**
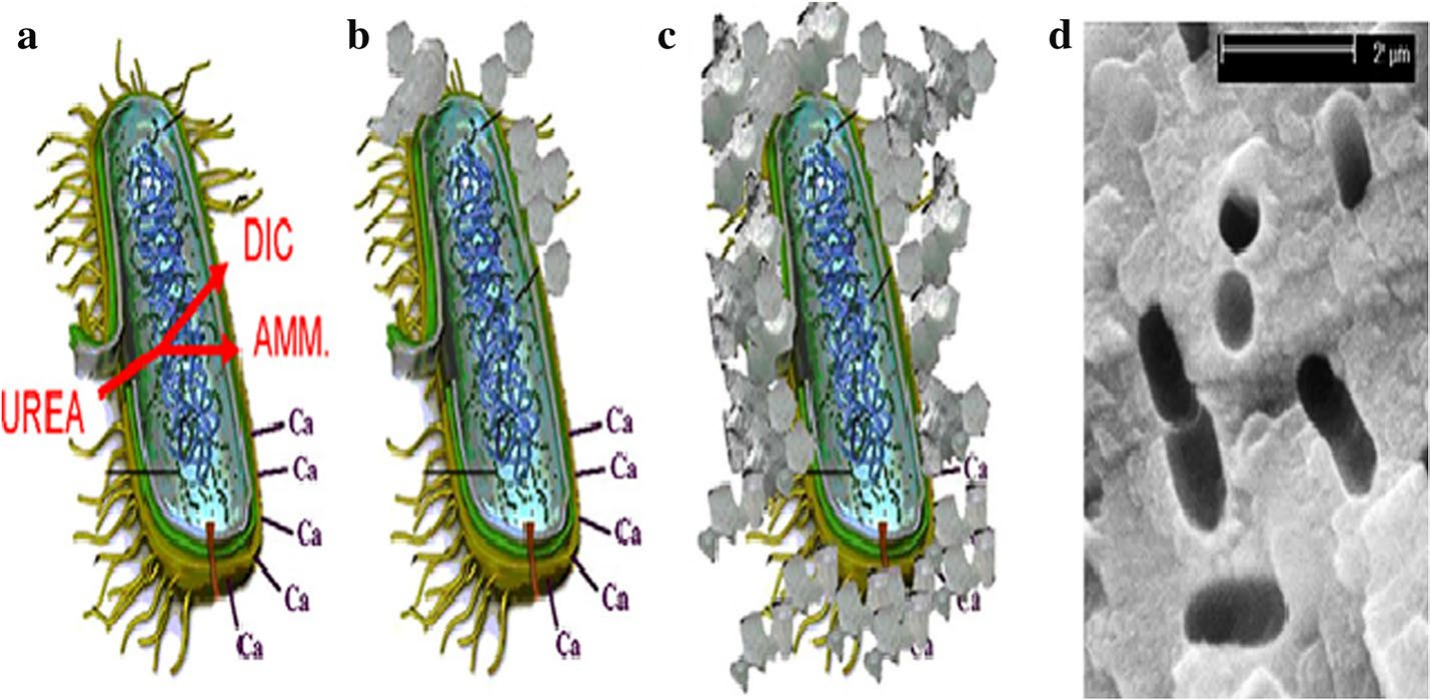
Ureolysis-driven calcite precipitation (*Source* from De Muynck et al. 2010)

The calcium carbonate (CaCO_3_) precipitation process is a straightforward and easily controllable mechanism of MICP that can produce high concentrations of CaCO_3_ in short period of time (Dhami et al. 2013a). Urease influences the chemical process associated with the formation of biominerals through four different parameters (Hammes and Verstraete 2002) such as pH, dissolved inorganic carbon (DIC) concentrations, calcium concentrations and the availability of nucleation sites. The first three parameters influence the carbonate ions concentration (CO_3_^2−^) (i.e., saturation state), while the last parameter (i.e., availability of nucleation sites) is very important for stable and continuous calcium carbonate formation (Phillips et al. 2013). In the biomineralization process, bacteria serve as nucleation sites, through which calcium carbonate precipitates with the bacteria. All of these parameters greatly affect either the ureolytic activity or CaCO_3_ crystal formation. Bacterial cell surfaces have negatively charged groups that act as scavengers for divalent cations (e.g. Ca^2+^, Mg^2+^) by binding them onto their cell surfaces at neutral pH, which make ideal nucleation sites for calcite deposition (Ferris et al. 1996; Stocks-Fischer et al. 1999; Ramachandran et al. 2001). However, Ca^2+^ ions can bind more frequently onto the negatively charged cell surface of bacteria than Mg^2+^ due to their having greater power for ionic selectivity (Wold 1994; Sanchez-Roman et al. 2007). Subsequently, the bound cation (metal ions) reacts with anions (carbonate) to form calcium carbonate in an insoluble form (Fig. 1). Bacterial cells are very important for the precipitation of CaCO_3_, because the bacteria provide nucleation sites (heterogeneous nucleation) and affect the specific types of minerals formed (Douglas and Beveridge 1998; Rodriguez-Navarro et al. 2012).

## Mechanism of calcite precipitation

Urease catalyzes the hydrolysis of urea into ammonium and carbonate. In this reaction, one mole of urea is hydrolyzed to one mole of ammonia and one mole of carbamic acid (Eq. 1), which is spontaneously hydrolyzed to another one mole of ammonia and carbonic acid (Eq. 2) (Stocks-Fischer et al. 1999; Burne and Chen 2000; Hammes et al. 2003a).

1 $${\text{CO}}\left( {{\text{NH}}_{2} } \right)_{2} + {\text{H}}_{2} {\text{O}}\mathop{\longrightarrow}\limits^{Microbialurease}{\text{NH}}_{2} {\text{COOH}} + {\text{NH}}_{3}$$

2 $${\text{NH}}_{2} {\text{COOH}} + {\text{H}}_{2} {\text{O}}\mathop{\longrightarrow}\limits^{Spontaneous}{\text{NH}}_{3} + {\text{H}}_{2} {\text{CO}}_{3}$$

These two products (NH_3_ and H_2_CO_3_) are further equilibrated in water to form bicarbonate (Eq. 3) and two moles of ammonium and two moles of hydroxide ions (Eq. 4). The hydroxide ions result in an increase of pH, which can shift the bicarbonate equilibrium, resulting in the formation of carbonate ions (Fujita et al. 2008) (Eq. 5). This shift can then precipitate the metal ions. The generation of NH_4_^+^ increases the local pH and the reaction continues spontaneously to form calcium carbonate (Ferris et al. 1996; Mitchell and Ferris 2005).

3 $${\text{H}}_{2} {\text{CO}}_{3} \leftrightarrow {\text{HCO}}_{3}^{ - } + {\text{H}}^{ + }$$

4 $$2{\text{NH}}_{3} + 2{\text{H}}_{2} {\text{O}} \leftrightarrow 2{\text{NH}}_{4}^{ + } + 2{\text{OH}}^{ - }$$

5 $${\text{HCO}}_{3}^{ - } + {\text{H}}^{ + } + 2{\text{OH}}^{ - } \leftrightarrow {\text{CO}}_{3}^{2 - } + 2{\text{H}}_{2} {\text{O}}$$

CaCO_3_ precipitation occurs at the bacterial cell surface if there are sufficient concentration of Ca^2+^ and CO_3_^2−^ in solution (Fig. 1) (Qian et al. 2010) (Eqs. 6, 7)

6 $${\text{Ca}}^{2 + } + {\text{Bacterial}}\;{\text{cell}} \to {\text{Cell-Ca}}^{2 + }$$

7 $${\text{Cell-Ca}}^{2 + } {\text{CO}}_{3}^{2 - } \to {\text{Cell-CaCO}}_{3}$$

## Different polymorphs and their effects in various calcium sources

Biomineralization can lead to produce different phases of CaCO_3_ anhydrous polymorphs such as calcite, aragonite and vaterite, as well as hydrated crystalline phases such as monohydrocalcite (CaCO_3_·H_2_O) and hexahydrocalcite or ikaite (CaCO_3_·6H_2_O) and amorphous calcium carbonate (ACC) (Krumbein 1979; Hammes et al. 2003a; Wei et al. 2003; Ben Chekroun et al. 2004; Xu et al. 2006; Chen et al. 2009; Sanchez-Navas et al. 2009; Gebauer et al. 2010; Dhami et al. 2013b). Among these, calcite and vaterite are the most common polymorphs (Gonzalez-Munoz et al. 2010; Rodriguez-Navarro et al. 2007; Dhami et al. 2013b, 2014). Vaterite is a minor, metastable and transitional phase during calcite formation (Tourney and Ngwenya 2009). Calcite is the most thermodynamically stable polymorph of CaCO_3_ and the primary product of CaCO_3_ in many MICPs (Spanos and Koutsoukos 1998; Stocks-Fischer et al. 1999; Okwadha and Li 2010; Ganendra et al. 2014). In contrast, Rivadeneyra et al. (1996) reported that aragonite is the predominant crystal formed by *Deleya halophila.* Chen et al. (2009) found that CaCO_3_ produced by *Proteus mirabilis* has an unusual morphology and structure, consisting of vaterite hollow spheres. Calcite and vaterite are different solid state phases of CaCO_3_. The precipitation of CaCO_3_ by mixing concentrated Ca^2+^ and CO_3_^2−^ solution involves at least three steps namely formation of amorphous calcium carbonate which is a form of CaCO_3_ with low stability and high solubility, transformation of amorphous CaCO_3_ into vaterite, and subsequent transformation of thermodynamically unstable vaterite into stable calcite (Spanos and Koutsoukos 1998; Wei et al. 2003; Shen et al. 2006; Hua et al. 2007). Pouget et al. (2009) have proposed the template controlled the initial stages of CaCO_3_ formation i.e. before transformation of ACC into vaterite and calcite. The initial stages of CaCO_3_ precipitation start with the formation of prenucleation clusters, aggregation of the clusters to form ACC nanoparticles and association of the nanoparticles with the template surface to initiate the ACC growth.

**Fig. 2:**
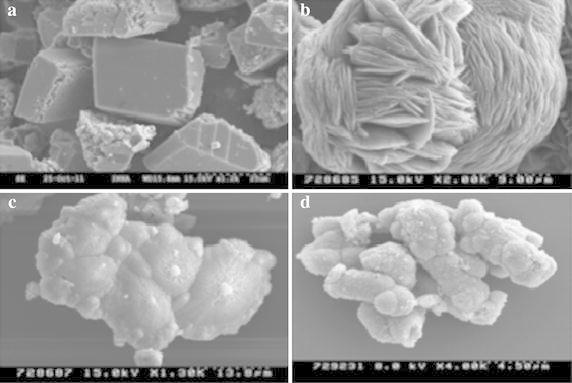
Scanning electron micrographs showing the effects of different calcium sources on the shape of the crystals formed. **a** Calcium chloride, **b** calcium acetate, **c** calcium lactate, **d** calcium gluconate (*Source* from Gorospe et al. 2013)

Different calcium sources induce crystal with different shapes (Fig. 2), with a rhombohedral shape induced by calcium chloride being characteristic of the most stable form of CaCO_3_ (calcite) (De Yoreo and Vekilov 2003; Favre et al. 2009; Gorospe et al. 2013). Other calcium sources also induced different shape of CaCO_3_ (Fig. 2). For example, calcium acetate induces a lettuce like or lamellar shape, (a metastable form of CaCO_3_) composed of vaterite, while calcium lactate and calcium gluconate induces a more complex form with packing that leads to growth of vaterite with a spherical shape (Tai and Chen 1998). The morphological differences in the crystal formation may be strain-specific, owing to differences in urease activity (Hammes et al. 2003a; Park et al. 2010). Alternatively, these differences could reflect the specific EPS protein produced by different bacterial types controlling calcite or aragonite polymorph selection (Kawaguchi and Decho, 2002), because EPS proteins may specifically bind Ca^2+^ and promote carbonate precipitation (Dhami et al. 2013b). The composition of the growth media or culture may also affect the crystal morphology because different bacterial species are able to precipitate different amounts, shapes and types of carbonate crystals from the same synthetic medium (Ferrer et al. 1988; Hammes and Verstraete 2002; Dhami et al. 2013b). Examination of the cells by electron microscopy reveals that calcium carbonate precipitation is closely associated with bacterial cells (Fig. 2).

**Fig. 3:**
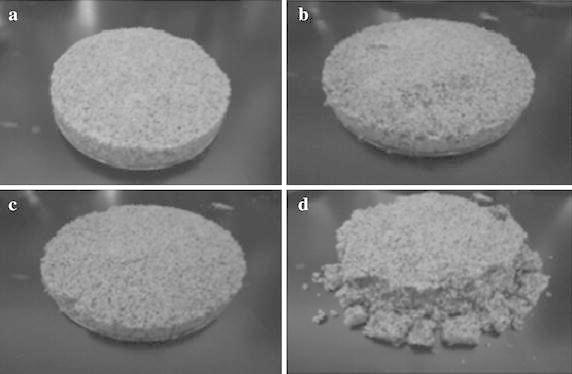
Bioconsolidated sand blocks made from different calcium salts. **a** calcium chloride, **b** calcium acetate, **c** calcium lactate, **d** control (*Source* from Gorospe et al. 2013)

**Fig. 4:**
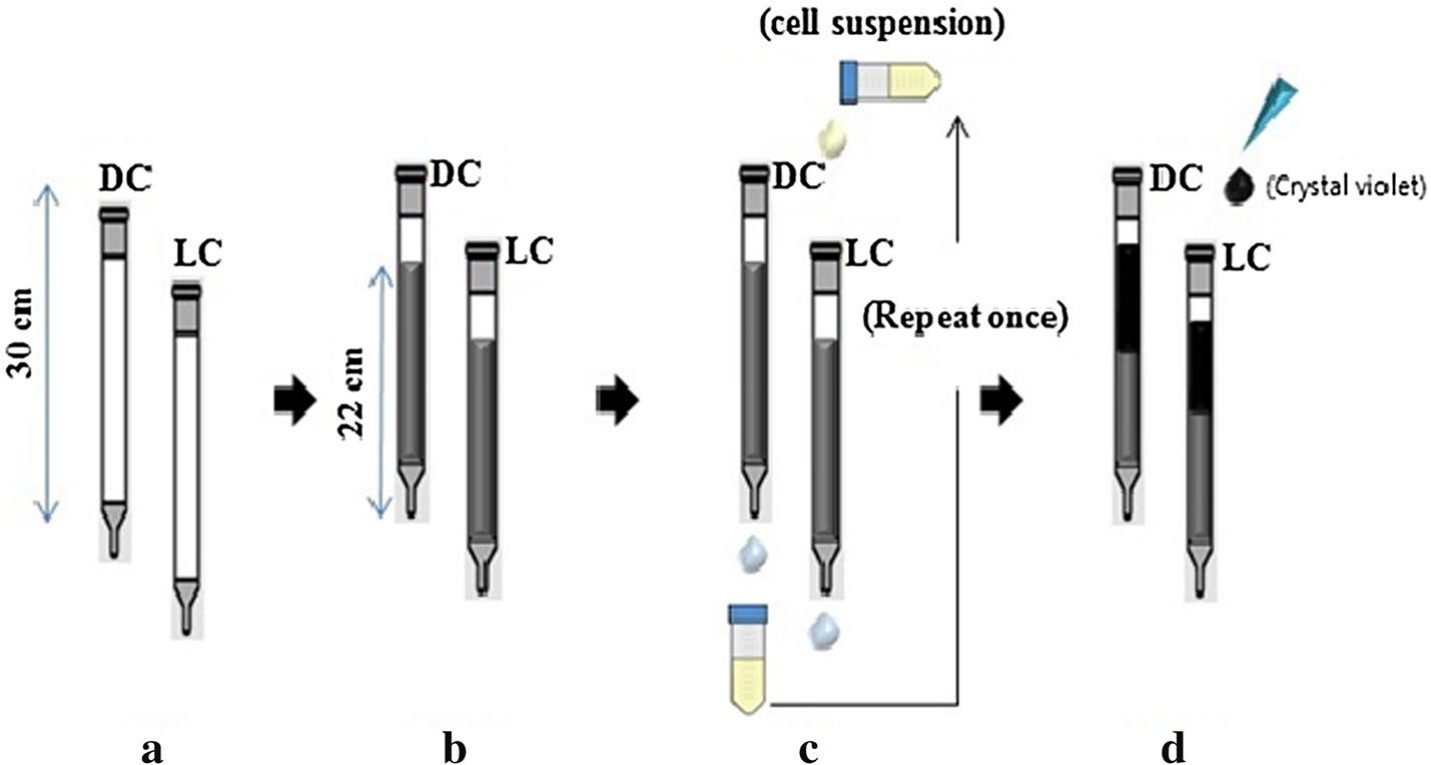
Impermeability tests. **a** A plastic pipette (25 ml) was used as a column, **b** sand slurry (50 g) was packed into the column, **c** cell suspension (10 ml) was passed through each column and flow-through was reloaded once, **d** crystal violet (2 ml) was dropped into a packed sand column to measure the migration distance (*Source* from Kang et al. 2014b)

Even though, many researchers have used different calcium sources for the induction of CaCO_3_ precipitation, calcium chloride is the best source for induction of calcite precipitation (Achal and Pan 2014). The effects of different calcium salts on stabilization of sand particles by bioconsolidation were investigated and blocks were formed in all samples treated by biocementation using various calcium sources (Fig. 3); however, the sand blocks collapsed after the Petridish demolded in the control samples, because dead cells were used (Gorospe et al. 2013). Our group has developed a simple method for testing impermeability to determine the efficiency of MICP. Specifically, the degree of impermeability was determined by measuring the migration distance of crystal violet. The calcium carbonate crystals are deposited between sand particles, resulting in plugging (Fig. 4).

## Factors affecting the efficiency of MICP

The activity of urease and the amount of CaCO_3_ precipitation are based on several environmental factors. Indeed, many factors affect urease activity and calcium precipitation including bacteria type, bacteria cell concentrations, pH, temperature, urea and calcium concentrations (Hammes and Verstraete 2002; Mortensen et al. 2011; Ng et al. 2012; Qabany et al. 2012).

### Bacteria type

The type of bacteria is essential to the production of urease capability. Many urease producing bacteria have been investigated, including *Aerobacter aerogenes*, *B. megaterium*, *B. subtilis*, *Bacillus* sp. CR2, *B. thuringiensis*, *D. halophila*, *Halmonas eurihalina*, *Helicobacter pylori*, *Kocuria flava* CR1, *L. sphaericus* CH5, *Methylocystis parvum*, *Myxococcus xanthus*, *Proteus mirabilis, Pseudomonas denitrificans*, *Spoloactobacillus* sp., *Sporosarcina ginsengisoli* and *Sporosarcina pasteurii* (Perez–Perez et al. 1994; Rivadeneyra et al. 1996, 1998; Stocks-Fischer et al. 1999; Ben Chekroun et al. 2004; Karatas et al. 2008; Chen et al. 2009; Achal et al. 2011, 2012b; Dhami et al. 2013b, 2014; Gorospe et al. 2013; Achal and Pan 2014; Ganendra et al. 2014; Kang et al. 2014a). Different types of bacteria were found to be able to produce various amounts of urease and calcium carbonate precipitation (Table 1). *Bacillus* group is a common type of bacteria used for the production of urease and calcite precipitation. For example, *Sporosarcina pasteurii*is the main organism used for multiple applications such as remediation of heavy metals and radionuclides, crack remediation in concrete and soil improvement (Whiffin et al. 2007; Sarada et al. 2009; Gorospe et al. 2013; Lauchnor et al. 2013; Li et al. 2013), while *B. megaterum* is used to enhance the concrete strength and durability of building materials and structures (Siddique et al. 2008; Soon et al. 2013; Dhami et al. 2014). *L. sphaericus* CH-5 and *K. flava* CR1 are used for the removal of cadmium and lead, respectively, from the environment (Achal et al. 2012a; Kang et al. 2014a). Achal et al. (2009a) developed a mutant strain of *Sporosarcina pasteurii* capable of producing more urease activity and CaCO_3_ precipitation than the wild strain of *Sporosarcina pasteurii* MTCC 1761.

### Bacteria cell concentration

High concentrations of bacterial cells (from 10^6^ to 10^8^ cells) increase the amount of calcite precipitation by MICP, via increases in the urease concentration for urea hydrolysis (Okwadha and Li 2010). Therefore, urea hydrolysis has a direct relationship with bacterial cell concentrations (Ng et al. 2012). Stocks-Fischer et al. (1999) reported that bacterial cells served as nucleation sites for CaCO_3_ precipitation, because the availability of the nucleation site is very important for calcite precipitations (Ng et al. 2012). Stocks-Fischer et al. (1999) compared the efficiency of microbially induced CaCO_3_ precipitation with chemically induced precipitation at pH 9.0 and confirmed that 98 % of the initial Ca^2+^ concentrations were precipitated microbially, but only 35 and 54 % precipitated chemically in water and medium, respectively. This difference occurred because the bacterial cells provide the nucleation sites for CaCO_3_ precipitation and create an alkaline environment for the induction of further growth of calcite (Stocks-Fischer et al. 1999).

### pH

Calcite precipitation is influenced by pH, because the urease enzyme will only be active at pH values specific for urea hydrolysis. Many investigators have reported that the optimum pH for urease is 8.0, above which the enzyme activity decreases (Stocks-Fischer et al. 1999; Gorospe et al. 2013). A high pH is very important for ammonia production by urea hydrolysis. Aerobic bacteria release CO_2_ via cell respiration, which is paralleled by an increase in pH due to ammonia production (Ng et al. 2012). If the pH levels become low, the carbonate will tend to dissolve rather than precipitate (Loewenthal and Marais 1978). Most calcite precipitation occurs under alkaline conditions from pH 8.7 to 9.5 (Stocks-Fischer et al. 1999; Ferris et al. 2003; Dupraz et al. 2009), but Mobley et al. (1995) found that the acid urease and optimum pH were nearly neutral. Stabnikov et al. (2013) recently investigated whether halophilic and alkaliphilic urease producing bacteria are active at high concentrations of inorganic salt and pH above 8.5 and the conditions suitable for manufacturing biocement.

### Temperature

Like other enzymatic reactions, the catalysis of urea by urease is temperature dependent. The optimum temperature for most ureases ranges from 20 to 37 °C (Mitchell and Ferris 2005; Okwadha and Li 2010) and the optimum range of the enzymatic reaction depends on environmental conditions and concentration of reactants in the system. Mitchel and Ferris (2005) reported that the urease activity increased by about 5 times and 10 times when the temperature increased from 15 to 20 °C and 10 to 20 °C, respectively. Ferris et al. (2003) investigated the kinetic rate of urease and the temperature dependence of ureolytic CaCO_3_ precipitation by *B. pasteurii* at 10 and 20 °C in artificial ground water. Dhami et al. (2014) found that urease was completely stable at 35 °C, but when the temperature increased to 55 °C the enzyme activity decreased by almost 47 %.

### Urea and Ca^+^ concentrations

The hydrolysis of urea by urease not only increases the pH, but also uses it as a nitrogen and energy source (Mobley and Hausinger 1989; Achal et al. 2009a). It is possible that individual microorganisms can produce ammonia as a result of enzymatic hydrolysis of urea to create an alkaline micro-environment around the cell and increase the pH, subsequently inducing the CaCO_3_ precipitation (Stocks-Fischer et al. 1999). Microbial cell surfaces have negatively charged and act as scavengers for cations, particularly Ca^2+^, in aquatic environments by binding them onto their cell surfaces; accordingly, microorganisms act as ideal crystal nucleation sites (Stocks-Fischer et al. 1999; Ramachandran et al. 2001). Therefore, the ideal calcium source and concentration is important for CaCO_3_ precipitation. However, high concentrations of urea and CaCl_2_ (above 0.5 M) decrease the efficiency of calcite precipitation (Okwadha and Li 2010), which increased efficiency was observed at low concentrations (0.05–0.25 M). De Muynck et al. (2010) reported that the best urea and CaCl_2_ concentrations for calcite precipitation are 0.5 and 0.25 M, respectively. Actually, the Ca^2+^ is not likely utilized by metabolic processes, but accumulates outside the cell, where it is readily available for CaCO_3_ precipitation (Silver et al. 1975). Okwadha and Li (2010) reported that the amount of CaCO_3_ precipitation depends more on Ca^2+^ concentrations than urea concentrations. Hammes et al. (2003a) investigated the importance of Ca^2+^ for urease activity and found that enzyme activity increased by tenfold in the presence of Ca^2+^ when compared than the absence of Ca^2+^. Recently, Achal and Pan (2014) studied the calcium precipitation from *Bacillus* sp. CR2 when different calcium sources were used in nutrient broth containing urea. Among the various calcium sources used, calcium chloride is best for the production of calcite as well as the higher urease activity.

## Isolation of ureolytic bacteria from various sources

The main task of the MICP technique is isolation and selection of potent urease producing bacteria. To promote ureolysis–driven calcite precipitation, the microorganisms should produce a sufficient amount of urease enzyme. Therefore, many investigators have isolated ureolytic microorganisms from various sources (Dejong et al. 2006; Hammes et al. 2003a; Achal and Pan 2014; Kang et al. 2014a) (Table 1). Ureolytic microorganisms that can induce CaCO_3_ precipitation have been studied for multiple applications such as remediation, consolidation and cementation (Ivanov and Chu 2008; De Muynck et al. 2010; Phillips et al. 2013).

Hammes et al. (2003a) reported that CaCO_3_ precipitating strains were isolated from garden and landfill soil from Ghent, Belgium. Achal et al. (2009a) developed a phenotypic mutant of *Sporosarcina pasterurii* by UV irradiation and compared it with the wild type strain MTCC 1761. The mutant strain (Bp-M3) was able to grow at higher pH (up to 11) than the wild type (up to 10) and to produce high urease activity and calcite precipitation. The increase in pH is very important to enhancement of the urease activity and calcium carbonate precipitation. The urease producing bacteria *Bacillus* sp. CR2 was isolated from mine tailing soil of Urumgi, Xinjiang, China (Achal and Pan 2014), and several *Sporosarcina* species were isolated from nursery garden soil at Tsinghwa University, China (Li et al. 2013). Positive strains were identified by the pink color formed upon hydrolysis of urea in urea test agar plates.

Dhami et al. (2013b) recently isolated the ureolytic bacterial strains from calcareous soil samples collected in Andhra Pradesh, India. Five positive strains (*B. megaterium, B. thuringiensis, B. cereus, B. subtilis* and *L. fusiformis*) were selected based on the urease activity, and calcite precipitation. However, *B. megaterium* produced highest urease activity (690 U/ml), and calcite precipitation (187 mg/100 ml). Other urease producing bacteria were isolated from an abandoned express way and abandoned mining sites in Gangwondo, Korea (Kang et al. 2014a), four of which showed urease activity and calcite production. However, only two strains of *Lysinibacillus* sp. were able to produce high urease activity and calcite precipitation. The same group isolated another strain of urease producing bacteria, *Sporosarcina pasteurii* WJ-2 from abandoned express way sites, in Gangwondo, Korea (Unpublished data). Sarada et al. (2009) screened three different microorganisms for urease production and found that *B. pasteurii* could produce urease at levels approximately two-fold higher than other tested microorganisms. Some investigators have also revealed ureolytic activity in situ in natural soil and ground water systems (Nielsen et al. 1998; Fujita et al. 2008; Tobler et al. 2011).

All bacteria could produce various amounts of urease and calcite precipitation (Table 1), while some microorganisms could produce high levels of urease and were involved in the hydrolysis of urea (Achal et al. 2009a; Dhami et al. 2013b, 2014). The endospore forming bacteria *Sporosarcina pasteurii* ATCC 11859 and *B. megaterium* have been shown to produce high levels of urease and have therefore been extensively studied. The urease activity was determined by phenol-hypochloride assay (Natarajan 1995). One unit of urease is defined as the amount of enzyme hydrolyzing 1 µmol urea/min. Bachmeier et al. (2002) investigated the role of nickel ions in the active site of the urease enzyme for its functional activity and the structural integrity of the enzyme. They also demonstrated recombinant transformation of the *B. pasteurii* urease gene to *Escherichia coli* HB101 containing pBU11plasmid. However, the amount of calcite precipitation by recombinant strain *E. coli* HB101 was lower than that of the wild type (Bachmeier et al. 2002).

## MICP applications

The MICP process is an effective and eco-friendly technology that can be applied to solve various environmental problems, including remediation of heavy metals and radionuclides, bioconsolidation, biocement, CO_2_ sequestration and other applications (De Muynck et al. 2010; Mitchell et al. 2010; Yoshida et al. 2010; Hamdan et al. 2011; Achal et al. 2012a, c).

## Removal of heavy metals and radionuclides

### Removal of heavy metals

Due to the rapid development of industrialization, heavy metal and radionuclide contaminants from industrial activities pose a major threat to the environment owing to their toxicity, non-biodegradability and persistent accumulation (Gazso 2001; Bahadir et al. 2007; Perez-Marin et al. 2008; Guo et al. 2010). Moreover, the heavy metals and radionuclides that accumulate in the environment create many health problems for humans and other living organisms. Some heavy metals are essential to human health in small quantities, but toxic in the large quantities released by industry (Guo et al. 2010; Fu and Wang 2011). For example, water sources contaminated by toxic heavy metals leaching from industrial wastes introduce heavy metals to plants, which are then ingested by animals that are subsequently consumed by humans. Indeed the ingestion of plant and animal based foods is the largest source of heavy metals exposure to humans (Radojevic and Bashkin 1999; Mulligan et al. 2001). The level of toxicity is based on the concentrations of the particular heavy metals; therefore, heavy metal ion contaminants are a very serious problem in the environment and their removal from contaminated soil and wastewater requires attention (Vullo et al. 2008).

Common heavy metals such as cadmium, chromium, cobalt, copper, arsenic, lead, nickel, selenium, silver, zinc, mercury, antimony, and thallium are naturally occurring, but become concentrated as a result of anthropogenic activities (Perez-Marin et al. 2008; Guo et al. 2010). Conventional treatments such as adsorption, chemical precipitation, electrochemical treatment, evaporation method, filtration, ion exchange, membrane technology, oxidation/reduction, and reverse osmosis (Kapoor and Viraraghavan 1995; Volesky 2001; Bai et al. 2008; Vullo et al. 2008; Wang and Chen 2009) have been used to remove heavy metals from contaminated environments. Unfortunately, these traditional methods often do not remove the metals successfully because they are ineffective, expensive, and consume high amounts of chemicals and energy (Fu and Wang 2011). In recent years, many biological treatments (using microorganisms) have been introduced to remove heavy metals from contaminated sites through phytoremediation, bioaccumulation, biocoagulation, bioleaching, biosorbents and bioimmobilization (Volesky 2001; Gadd 2000; Gazso 2001; Lloyd and Lovely 2001; Lin and Lin 2005; Achal et al. 2011). However, these methods are also not effective because they are expensive, time consuming and result in the release of immobilized or adsorbed heavy metals back to the environment (Achal et al. 2011). Therefore, alternative methods such as MICP are needed to remove the heavy metals effectively, economically and in an eco-friendly manner (Hamdan et al. 2011; Achal et al. 2012a, b). Several authors have reported that MICP has the potential to remediate heavy metals and radionuclides from the environment (Hamdan et al. 2011; Achal et al. 2012c).

**Fig. 5:**
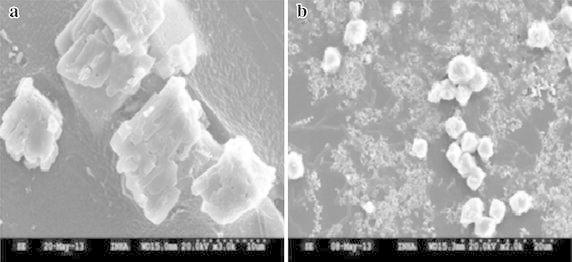
Scanning electron micrograph showing the remediation of cadmium by *L. sphaericus* CH-5. **a** CdCl_2_, **b** CdCO_3_ (*Source* from Kang et al. 2014a)

Heavy metal toxicity affects the microbial growth and efficiency of MICP; therefore, several researchers have isolated metal tolerant microbes with ureolytic capability from various environments to improve the efficiency of the MICP process (Guo et al. 2010; Achal et al. 2011; Kang et al. 2014a). In MICP process, calcites can be incorporated heavy metals (e.g., Pb^2+^) onto their surfaces via substitution of suitable divalent cations (Ca^2+^) in the calcite lattice (Eq. 8), after which these compounds are changed from soluble heavy metals to insoluble forms i.e., detoxify the heavy metals (Pan 2009; Achal et al. 2011). Li et al. (2013) reported that a few species of *Sporosarcina* and *B. lentus* urease producing bacteria were able to remove 88 to 99 % of heavy metals after 48 h of incubation. SEM analyses showed evidence of the transformation of heavy metals into stable calcite and other crystal forms (Figs. 2, 5).

8 $${\text{Pb}}^{2 + } + {\text{OH}}^{ - } + {\text{HCO}}_{3}^{ - } = {\text{PbCO}}_{3} + {\text{H}}_{2} {\text{O}}$$

#### Copper

Copper is naturally present in soil, air and water and an essential micronutrients for cell functions, but the ingestion of large quantities of copper has serious toxicological effects (Paulino et al. 2006; Achal et al. 2011). Achal et al. (2011) isolated the copper tolerant bacteria *K. flava* CR1 from a mining area in China, using the MICP process and achieved about 97 % copper removal from the environment. Moreover, they found that the bacteria had high levels of urease activity, which was involved in removal of high concentrations of copper. The high concentration of copper primarily affects the bacterial growth, but it improves the copper removal rate by *K. flava* CR1 (Achal et al. 2011). Recently, Li et al. (2013) reported that the removal rate of copper by *Sporosarcina koreensis* UR47 was approximately 93 %, which was higher than that by *Sporosarcina pasteurii* ATCC 11859.

#### Cadmium

Cadmium is an extremely toxic metal commonly used in electroplating, in industrial paints, manufacture of batteries, construction and agriculture. Kang et al. (2014a) investigated the potential removal rate of cadmium (99.5 %) by MICP after 48 h in beef extract, peptone and urea (BPU) media (Fig. 5). Ma et al. (2009) reported that water borne heavy metals (Cu, Zn and Cd) were removed well by CaCO_3_-dominated red mud. Recently, another study also found that *Terrabacter tumescens* removed more than 99 % of the cadmium from soil wastewater via MICP (Li et al. 2013).

#### Chromium

Chromium is common environmental pollutant in the environment, although trace levels of some forms of chromium such as Cr(III) in food and water and appear to be benign. However, Cr(VI) is highly mobile, toxic, soluble and carcinogenic (Kamaludeen et al. 2003). Many investigators have attempted to remove chromium from Cr-contaminated soil and water by phytoremediation and bioremediation (Chandra et al. 1997; Lytle et al. 1998; Jeyasingh and Philip 2005); however, these techniques release the immobilized or adsorbed heavy metals back into the soil or water. Using the MICP process, chromate can interact with CaCO_3_ in co-precipitated form and effectively remove chromium from Cr-contaminated sites (Hua et al. 2007; Achal et al. 2013). A few studies have shown the role of metal ions during the transformation process (Nassrallah-Aboukais et al. 1999; Wei et al. 2003). The process of transformation from vaterite to calcite, which is important to produce stable crystals, occurs via a spontaneous reaction through either simple contact with water or heating. Accordingly, if there are any metal ions on the surface of the vaterite, the transformation process may be delayed or stabilized (Nassrallah-Aboukais et al. 1999). However, the same authors reported that the transformation process is not delayed in the presence of Cu^2+^ on the surface of the vaterite. In contrast, Hua et al. (2007) reported that the transformation process was delayed or inhibited in the presence of Cr(VI).

#### Lead

Lead is the most prevalent heavy metal contaminant among environmental pollutants (Di Maio 2001). Removal of lead by currently available remediation methods such as biosorption and other techniques is ineffective and requires high volumes of reagents. Additionally, these methods are expensive, result in generation of toxic sludges and require a means of safe disposal (Suh et al. 1998; Pan et al. 2005; Wang and Chen 2009). Puyen et al. (2012) found that the lead and copper removal rate were 36.07 and 25.42 %, respectively, in response to the biosorption method in culture media by heavy metal tolerant *Micrococcus luteus* DE2008. In the MICP process, lead was bound with the MICP product (calcite), which was responsible for Pb immobilization and resulted in significantly reduced Pb levels in the environment (Achal et al. 2012a; Kang et al. 2015a). Very recently, Li et al. (2013) found that the potential removal rate of lead by *Sporosarcina koreensis* UR47 was nearly 100 %.

#### Arsenic

Arsenite (AsIII) is the most common and toxic arsenic species. Arsenite is highly mobile in soil and easily leached into groundwater (Achal et al. 2012b). Many researchers have used various bioremediation methods to remove arsenic from the environment (Yamamura et al. 2003; Fayiga et al. 2004; Kirk et al. 2004); however, these methods are ineffective because the immobilized or adsorbed heavy metals are again released into the environment. Achal et al. (2012b) isolated an arsenic tolerant bacteria *Sporosarcina ginsengisoli* CR5 and reported that its growth decreased when arsenic was present in the media. However, the strain was able to effectively remove about 96.3 % of the arsenic after 7 days of cultivation in NBU media containing 50 mM As(III). Another study reported that about 96.9 % of arsenic was removed from aqueous media containing only 40 mM of As(III) (Aksornchu et al. 2008). The main advantage of using MICP for arsenic removal is that the product (carbonate metal complex) is insoluble and traps the arsenic, preventing it from being released back into the environment (Achal et al. 2012b).

### Removal of radionuclides

The disposal of radionuclide wastewater from commercial nuclear plants is a major issues associated with nuclear waste management because it is highly toxic to the environment, particularly to human health (Ahmadpour et al. 2010). Many researchers have used various physico-chemical processes to remove radionuclides such as chemical precipitation, flocculation, ion exchange, membrane process, immobilization and adsorption (Mishra and Tiwary 1999; El-Kamash et al. 2006; Rawat et al. 2006; Rout et al. 2006; Omar et al. 2009). Fujita et al. (2008) investigated a pump and treat method, but found that it was ineffective at removal of radionuclides from the contaminated environment. Therefore, an alternative method of MICP involved cleaning up the radionuclides safely from the environment. The MICP method stimulates ureolytic microorganisms to promote CaCO_3_ precipitation, which in turn leads to promote co-precipitation of radionuclides by substitution of Ca^2+^ ion and formation of radionuclide carbonate minerals (Mitchell and Ferris 2006; Fujita et al. 2010).

#### Strontium

In living organisms, strontium is highly toxic and soluble; therefore, it can be readily passed through the food chain from contaminated soil or water. Additionally, strontium is capable of exerting long term health impacts due to its long half-life (28.8 years) (Singh et al. 2008). The mobility and carcinogenic effects of Sr affect groundwater usability (Lauchnor et al. 2013), and the conventional remediation techniques are expensive and ineffective (AbdEl-Sabour 2007). Strontium 90 exists in the environment as the Sr^2+^ ion, which has chemical similarity to Ca^2+^; therefore, Sr^2+^ can replace calcium ions in living systems (Singh et al. 2008; Achal et al. 2012c). Many researchers have successfully demonstrated the co-precipitation of ^90^Sr^2+^ into calcite by substituting Ca^2+^ in calcite crystal through MICP effectively (Fujita et al. 2004; Smith et al. 2004; Mitchell and Ferris 2005; Achal et al. 2012c; Brookshaw et al. 2012). Warren et al. (2001) found that 95 % of strontium was captured in the solid phase by MICP when *Sporosarcina pasteurii*was used. Achal et al. (2012c) isolated the Sr-resistant and urease producing bacteria *Halomonas* sp. SR4, and reported approximately 80 % Sr removal from the soluble exchangeable fraction of aquifer quartz sand. Kang et al. (2014b) recently reported a similar removal rate of Sr from the soluble fraction of sand by *Sporosarcina pasteurii* WJ-2.

### Bioconsolidation of soil and sand

In geotechnical engineering, bioconsolidation is involved in prevention or stabilization of erosion and increasing slope stability. Conventional techniques such as applying cement or chemicals are primarily used to improve soil; however these can lead to permanent soil and water contamination or air pollution (Khodadadi and Bilsel 2012). Additionally, these synthetic chemicals can be injected into the subsurface to bind sand grains together, increasing soil strength and stiffness. However, this method is expensive, difficult to distribute uniformly and introduces hazardous substances into the soil (DeJong et al. 2010). New construction on weak soil results in low strength and high compressibility (Huat 2006; Ng et al. 2012). Many investigators have successfully applied chemical grout to improve the soil (Karol 2003; Basha et al. 2005); however, the pH of the soil was modified, soil and ground water were contaminated, and the toxicity of the soil increased (DeJong et al. 2006). Therefore, an effective technique to improve soil quality is necessary. MICP has been investigated as an attractive method of grouting to improve the soil structure. Whiffin et al. (2007) reported that sand stabilization treatment to increase the bacterial adhesion to sand, before MICP treatment and reduction of porosity and improvement of strength of soil found positively in sand packed columns after MICP treatment.

The induction of CaCO_3_ precipitation binds sand grains together at the particle–particle contacts, which increases the strength and stiffness of the soil (DeJong et al. 2010; Mortensen et al. 2011; Gorospe et al. 2013). This method utilizes biochemical processes to improve the engineering properties of soil such as shear strength and impermeability (Chu et al. 2012; Kang et al. 2015b). Ivanov and Chu (2008) compared the cost of conventional chemical grouting with microbial grouting and found microbial grouting ($0.5–9/m^3^ of soil) to be significantly less expensive than chemical grouting ($2–72/m^2^ of soil). The application of bioconsolidation can lead to a tenfold change in the primary properties of the sand such as permeability, stiffness, compressibility and shear strength (DeJong et al. 2010). MICP treatment has contributed greatly to improvement of the engineering properties of residual soil, but its applicability to soil types other than sand is still very limited (Soon et al. 2014). In addition, MICP improved the durability of construction and cementation materials.

**Fig. 6:**
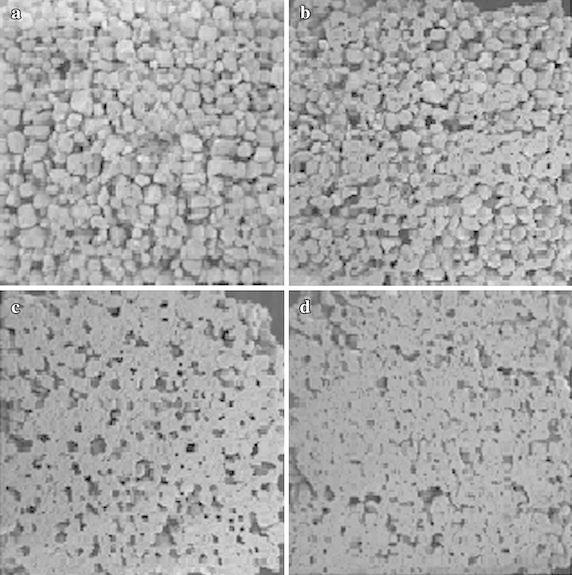
Scanning electron micrograph showing the bioconsolidated soil from *L. sphaericus* WJ-8. **a** Water, **b** YAU media, **c** vegetative cell only, **d** vegetative cell and spore. (Unpublished data)

The chemical grouting technique is not only expensive, but also requires many injection wells for treatment of large volumes. Using MICP, the reagents and catalysts are injected and transported to the location at which strengthening is required (Dhami et al. 2013a). Many researchers have reported improvement of shear strength and reduction in permeability of soil in response to ureolytic bacteria (DeJong et al. 2006; Whiffin et al. 2007; Chu et al. 2012). The CO_3_^2−^ ions precipitate with Ca^2+^ as calcite crystal, which generates cementing bridges between sand particles (Figs. 1, 3). The plugging of the soil restricts the water flow through the soil and then reduces the permeability. The calcite crystals formed between the soils particles along with embedded bacteria suggest that bacteria served as nucleation sites during the mineralization process (Stocks-Fischer et al. 1999; Ng et al. 2012). An increase of pores plugged due to the reaction of calcite was observed in the presence of vegetative cells and spores of *Lysinibacillus sphaericus* WJ-8 of soil (Fig. 6. Unpublished data).

### Bioconcrete or biocementation

Cement is widely used as a construction material to strengthen soil (Stabnikov et al. 2013). However, the production of cement has environment impacts during all stages of manufacturing. Additionally, global cement production accounts for about 5 % of the total industrial energy consumption and 5 % of anthropogenic CO_2_ emissions (Worrel et al. 2001). Chemical grout, which can be used in place of cement to strengthen soil, employs sodium silicate, calcium chloride, calcium hydroxide, acrylates, and acrylamides to bind soil particles (Karol 2003; Ivanov and Chu 2008). However, this method is expensive and toxic to humans and the environment (Karol 2003; DeJong et al. 2006; Ivanov and Chu 2008). Therefore, economical alternatives to chemical grouting are necessary. Biocement is an alternative to cement and chemical grouts (De Muynck et al. 2010; Stabnikov et al. 2011) that can produce binder materials via MICP treatment to seal fractures and improve the strength and durability of cementious materials (Phillips et al. 2013; Dhami et al. 2014). Biocementation of MICP has been applied to strengthen soil and treat the cracks in concrete (Ramachandran et al. 2001; De Muynck et al. 2008). Soil cementation materials include carbonates, hydroxides, phosphates, silicates and sulfides (Ivanov and Chu 2008). Calcium carbonate is primarily used in biocementation because it is commonly found in nature. Biocement can improve soil shear strength through the production of soil particle-binding materials in response to the introduction of bacteria and cementation reagents into the soil (Ng et al. 2012). Different bacterial strains have been shown to produce various levels of urease activity ranging from 2.2 to 20 mM of hydrolyzed urea/min (Harkes et al. 2010; Stabnikov et al. 2013). Urease activity should not be too high or too low for successful biocementation because urease activity in the range of 4.4 to 9.5 mM hydrolyzed urea/min. increased the strength of biocemented soil. Qian et al. (2009) reported that stronger aggregates of calcium carbonate formed at a low rate of urea hydrolysis. Following MICP treatment (by BHI cured bricks), the absorption of water by bricks cured in the media was lower (−14 %) than that of the control (25 %) because pores were blocked by calcite deposition, which prevented water and other pollutants from penetrating into the body of the bricks (Sarada et al. 2009). Ramachandran (2007) found that incorporation of high concentrations of bacterial cells into the cement mixture reduced compressive strength owing to inference of the biomass with the integrity of the mortar. However, other researchers reported that the compressive strength improved after MICP treatment relative to a control (De Muynck et al. 2008; Chahal et al. 2012).

The cracks form in concrete due to aging and freeze thaw cycles; however, many researchers have reported the remediation of cracks by MICP of *B. pasteurii* and other *Bacillus* species (Ramachandran et al. 2001; Achal et al. 2013). Bioclogging of soil restricts water flow through soil and reduces its permeability. The permeability of soil was reduced significantly through accumulation of biomass and production of exopolymeric substances (Vandevivere and Baveye 1992; Ng et al. 2012). DeJong et al. (2010) reported a reduction of pore size, porosity, and permeability, as well as improvement of the stiffness and strength of the porous media matrix in response to MICP. Bernardi et al. (2014) recently reported the manufacture of bio-bricks by MICP and compared the effectiveness with that of conventional cement and lime treated bricks. Actually, the type of CaCO_3_ polymorphs is important to the construction purpose because it has high stability and consolidating effects (Rodriguez-Navarro et al. 2003; Ganendra et al. 2014). The growth of *B. pasteurii* is affected by very high pH (above 12), particularly in concrete, because the optimum pH for the best growth of this strain is 9.0. For this instance, Bang et al. (2001) investigated an immobilization technique utilizing polyurethane (PU) for remediation of concrete cracks to protect the bacterial cells from the high pH of the concrete. However, during whole cell immobilization in PU, the viability of the cells encapsulated in PU polymer is uncertain (Wang and Ruchenstein 1993; Bang et al. 2001). Nevertheless, Bachmeier et al. (2002) suggested that the immobilized enzyme could overcome the loss of viability of whole cells in PU and that the immobilized enzyme will be environmentally safer than immobilized microorganisms. MICP is also involved in protection of concrete surfaces against the ingress of deleterious substances (e.g., chloride ions) (De Muynck et al. 2008).

### CO_2_ sequestration

Global warming is a major environmental issue occurring primarily in response to increasing concentrations of CO_2_ in the earth’s atmosphere (Yadav et al. 2011). Currently, the concentration of CO_2_ in the earth’s atmosphere is about 400 ppm; however, this is increasing at approximately 2 ppm/year (Source from Wikipidia). Thus, there is an urgent need to reduce the release of CO_2_ into the environment. The increasing atmospheric CO_2_ levels are mainly due to the burning of fossil fuels for energy production and consumption and other activities such as cement production and tropical deforestation (Malhi and Grace 2000; Goel 2010). CO_2_ is primarily removed from the atmosphere via photosynthesis by plants and marine organisms, while it is returned to the atmosphere via respiration by animals and chemoorganotrophic organisms. Many investigators have used different mechanisms to capture and dispose of CO_2_ in an environmentally safe manner (Sharma and Bhattacharya 2010). The most effective way to lower CO_2_ emissions into the environment is to reduce fossil fuel consumption. In nature, CO_2_ is sequestered by chemical fixation of CO_2_ in the form of carbonate such as calcite, aragonite, magnesite and dolomite, but the reaction rate is very slow (Mann 2001; Dhami et al. 2013a). Many researchers have proposed an alternative method of biological sequestration of CO_2_ by carbonic anhydrase (CA) enzyme to reduce atmospheric CO_2_ (Ramanan et al. 2009; Yadav et al. 2011). CA is a zinc containing metallo-enzyme that catalyzes the reverse hydration of CO_2_ to bicarbonate and is ubiquitous in prokaryotes and eukaryotes. In eukaryotes, CA is involved in many biochemical and physiological process such as photosynthesis, respiration, CO_2_ and ion transport and acid base balance (Karlsson et al. 1998; Tripp et al. 2001; Zhang et al. 2011; Dhami et al. 2014).

MICP is an effective method for the removal of CO_2_ from the environment (Ferris et al. 1994; Mitchell et al. 2010). In this method, CO_2_ is converted into carbonate minerals that can form different crystals such as calcite, aragonite, dolomite and magnesite. This method is safer and more eco-friendly than conventional methods of sequestering CO_2_ from the atmosphere. Bond et al. (2001) reported positively the transformation of CO_2_ to bicarbonate in the presence of Ca^2+^ in artificial sea water and a rapid decrease in CO_2_ concentration and increase in CaCO_3_ synthesis with CA enzyme. Liu et al. (2005) and Kim et al. (2012) found that the bovine CA and recombinant CA from *Nisseria gonorrhoeae* (NCA), rapidly catalyzed the reversible hydration of CO_2_ to bicarbonate and calcite crystal formations. Several studies confirmed that CO_2_ could be effectively sequestered into carbonate by CA from different organisms (Bond et al. 2001; Ramanan et al. 2009). Ramanan et al. (2009) investigated whether the addition of CA enzyme to reaction mixtures containing CaCl_2_ solution saturated with CO_2_, resulted in enhanced deposition of carbonate/bicarbonate salts. They also compared carbonate deposition by crude enzyme with purified enzyme. The purified enzyme was able to deposit almost 15 times more carbonate than crude enzyme (Ramanan et al. 2009). Most sequestration studies conducted to date have been based on the assumption that CO_2_ must first be separated from the exhaust gas produced by fossil-fuel combustion and then disposed of in depleted oil and gas wells, deep saline aquifers in the ocean or through deposition into minerals (Dupraz et al. 2009). Using this approach, CA enzymes are able to capture and fix anthropogenic CO_2_ into solid carbonate (CaCO_3_), resulting in production of a stable eco-friendly benign product (Ramanan et al. 2009).

**Fig. 7:**
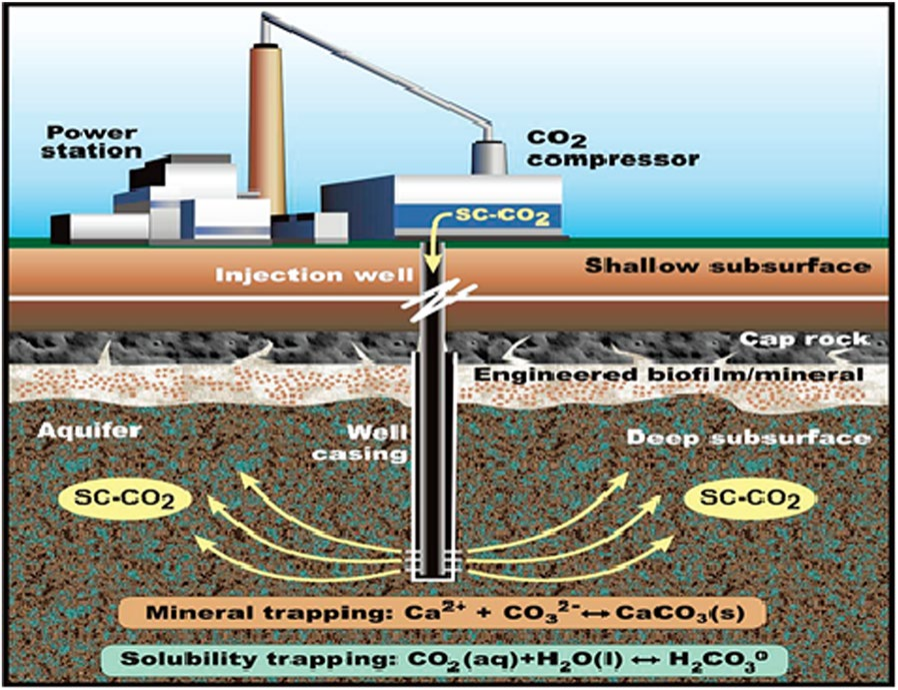
Schematic diagram of microbially enhanced carbon capture and storage (*Source* from Mitchell et al. 2010)

The geological sequestration of CO_2_ has also been accomplished by the injection of supercritical CO_2_ (SC-CO_2_) into deep geological environments, oil bearing formations, deep-seated coal beds and deep saline aquifers (White et al. 2003; Haszeldine et al. 2005; Mitchell et al. 2010). The critical over-saturation values necessary for CaCO_3_ precipitation have been discussed by many researchers (Ferris et al. 2003; Dupraz et al. 2009). These environments are known to shelter extensive and active microbial communities that could possibly interact with the injected CO_2_ (Amend and Teske 2005; Dupraz et al. 2009) (Fig. 7). Among many carbon capture and storage technologies, biotechnology using CA in an immobilized enzyme reactor at these plants holds great promise because it is viable and environmentally benign (Liu et al. 2005), and the generated carbonate minerals are safe methods of long term CO_2_ storage.

### Other applications

The preparation of useful products based on the integration of CO_2_ fixation and biomass utilization is important to future development of environmental engineering strategies. In material engineering, environmentally friendly technologies are required for the production of materials and composite with minimum levels of energy consumption, resource depletion and despoilment (Wakayama et al. 2005). The MICP method is an alternative method for the production of several materials. The biominerals of calcium carbonate or calcium phosphate are involved in production of complex multifunctional composites with organic macromolecules at extreme temperature and pressure (Wakayama et al. 2005). Particularly, calcite formed by *Geobacillus thermoglucosidasius* has many advantages when applied as filler in rubber and plastics, fluorescent particles in stationary ink and stationary markers for western blotting and other biochemistry applications (Ling et al. 2009; Yoshida et al. 2010).

Polychlorinated biphenyls (PCBs) containing oils comprise a serious environmental concern with the potential to impact human health. There are several methods available for removal of PCBs contaminated oil from the environment including solvent washing and hydroblasting followed by encapsulation in epoxy coatings, but these methods are ineffective for the removal of PCBs successfully. Alternatively, MICP is able to produce a coating to seal PCBs-contaminated areas. Indeed, MICP coated areas showed no leaching and a reduction in permeability of 1–5 orders or magnitude (Okwadha and Li 2011).

High concentrations of calcium ions in industrial wastewater are problematic because of the clogging of pipelines, boilers and heat exchangers through scaling or malfunctioning of aerobic and anaerobic reactors (Yu et al. 2001; Hammes et al. 2003b). MICP is an alternative technology for removal of inorganic contaminants from the environment, and many researchers have reported the use of MICP methods for removal of calcium from industrial wastewater (Van Langerak et al. 1997; Hammes et al. 2003a, b). Van Langerak et al. (1997) reported the removal of calcium from industrial wastewater using a fluidized sand bed calcification reactor that employed the alkalinity generated by microbes in a standard up-flow anaerobic sludge bed reactor. Using the biocatalytic calcification reactor, approximately 85–90 % (w/v) of the soluble calcium was precipitated as calcium carbonate and successfully removed through sedimentation in the treatment reactor (Hammes et al. 2003b). Therefore, this is an effective, eco-friendly and simple method for removal of calcium from industrial wastewater.

### Limitations and perspectives regarding MICP

Most potential applications of MICP technology in various fields have been discussed. However, there are a few limitations to the use of MICP that must be overcome before it can be widely applied on a commercial scale.

1. MICP may not be completely environmental friendly, because ammonium and nitrate are formed during the ureolysis-driven process, which can be toxic and hazardous to human health and soil microorganisms at high concentrations (Van Paassen et al. 2010). Additionally, ammonium present inside building materials can be nitrified into nitric acid by bacteria. This can in turn react with calcite to form calcium nitrate, which is a highly soluble component that contributes to bio-deterioration of building materials (Ganendra et al. 2014). Ganendra et al. (2014) recently investigated the MICP process (i.e., formate oxidation-driven CaCO_3_ precipitation) using calcium formate produced by *Methylocystis parvus* OBBP. They found that it was advantageous over ureolysis-driven processes because the calcium formate did not release the ammonia to the air or produce nitric acid when applied to building materials, resulting in decreased risk of pollution and bio-deterioration of the materials.
2. Another disadvantage of MICP is that microbial processes are usually slower and more complex than chemical process. This is because microbial activity is completely dependent on environmental factors such as temperature, pH, concentration of donors and acceptors of electrons, and concentration and diffusion rates of nutrients and metabolites. (Ivanov and Chu 2008). Therefore, it is difficult to apply MICP on a commercial scale.
3. The economic limitations to use of laboratory grade nutrient sources in field applications must be overcome. To address this, identification of alternative inexpensive nutrient sources for MICP technology is necessary. For example, corn steep liquor or lactose mother liquor may provide less expensive nutrient sources for successful commercialization (Achal et al. 2009b; Mitchell et al. 2010; Dhami et al. 2013a; Phillips et al. 2013). In addition to this limitation, the production of large volumes of reactants and bacterial cultures make MICP economically challenging when compared to traditional methods. Finally, additional investigations to improve the technology and reduce unwanted byproducts are needed to enable use of MICP on a commercial scale.

## Conclusions

A wide variety of microorganisms can be used in the production of urease for ureolysis-driven processes. However, microorganisms such as *Sporosarcina pasteurii* and *B. megaterium* have the greatest potential for use in MICP. Many researchers have developed various conventional methods for environmental clean-up, but these methods are inefficient and expensive. MICP has emerged as an effective and ecofriendly method for environmental remediation. MICP is used in various fields to remediate heavy metals and radionuclides from contaminated environments and for sequestration of atmospheric CO_2_. In addition, the same technology can be used to improve soil and sand quality, as well as cement sealing of concrete. MICP applications are not limited and are useful to other applications to produce safe and environmentally stable products. Even though the MICP process has many merits, further study is needed to overcome the limitations to use of this technology prior to its commercialization.
